# Prenatal stress and offspring depression in adulthood: The mediating role of childhood trauma

**DOI:** 10.1016/j.jad.2021.10.019

**Published:** 2022-01-15

**Authors:** Yiwen Liu, Jon Heron, Matthew Hickman, Stanley Zammit, Dieter Wolke

**Affiliations:** a Department of Psychology, University of Warwick, Coventry CV4 7AL, United Kingdom; b Population Health Sciences, Bristol Medical School, University of Bristol, United Kingdom; c MRC Centre for Neuropsychiatric Genetics and Genomics, School of Medicine, Cardiff University, United Kingdom; d Division of Health Sciences, Warwick Medical School, University of Warwick, United Kingdom

**Keywords:** Depression, Childhood trauma, Maternal depression, Family adversity

## Abstract

**Background:**

There is repeated evidence for a prenatal programming effect for the development of offspring depression. However, examination of environmental influences along this pathway is sparse. This study aimed to investigate the direct and indirect effects of pre- and postnatal stress on offspring depression in adulthood, via increased exposure to childhood trauma.

**Methods:**

A large longitudinal population-based cohort (*N* = 3506) was followed up from birth and assessed at 24 years. Diagnosis of depression was derived using the International Classification of Diseases–10th revision (ICD-10). Two separate sources of pre- and postnatal stress were examined – maternal depression and family adversity, and childhood trauma was assessed prospectively across childhood until 17 years.

**Results:**

Both pre- and postnatal maternal depression and family adversity were associated with offspring depression at 24 years in simple logistic regression models. When all pathways were modelled simultaneously, only childhood trauma was directly associated with offspring depression, and mediated all pathways from both sources of pre- and postnatal stress to offspring depression (7–16% of the total effect mediated). Sensitivity analysis on specific trauma found stronger evidence for a mediated pathway via physical, emotional abuse and peer bullying, compared to emotional neglect, sexual abuse and domestic violence.

**Conclusions:**

These findings indicate that reducing childhood trauma could be a target to decrease depression in the general population, and the focus should also be on families at high risk of experiencing pre- or postnatal stress, to provide them with better support.

## Highlights

• Childhood trauma was associated with increased risk of depression in adulthood.
• Prenatal and postnatal stress, indicated by maternal depression and family adversity, were associated with increased exposure to childhood trauma.
• Childhood trauma mediated all pathways from pre- and postnatal stress to offspring depression in adulthood, even after accounting for genetic risks.

## 1 Introduction

There is substantial evidence in the literature that adverse prenatal environment is associated with diseases and mortality in adulthood (Lewis et al., 2015). This has been formulated into the foetal programming hypothesis, which postulates that intrauterine adversity, for example growth restriction, can alter the biological systems of the developing child and increase their susceptibility to future diseases (Barker, 2007; O'Donnell and Meaney, 2016; Räikkönen and Pesonen, 2009; Van den Bergh et al., 2017). More recently the theory has been applied to the study of mental health disorders such as depression, and other sources of intrauterine adversity have also been investigated, in particular maternal stress in the prenatal period (O'Donnell and Meaney, 2016; Räikkönen and Pesonen, 2009).

Prenatal stress is often indicated by maternal mental health (e.g. depression) and family adversity (e.g. social and economic difficulties) in the literature (Lereya and Wolke, 2013). Although closely associated with each other, both have been independently associated with offspring outcomes and can be considered as two separate sources of stress (Lereya and Wolke, 2013), one from psychological and one from social/economic stress. There is strong evidence that prenatal maternal depression is associated with offspring depression in adulthood (Rogers et al., 2020; Tirumalaraju et al., 2020). Prenatal family adversity such as financial or relationship difficulties have also been associated with offspring depression even after accounting for maternal mental health (Kingsbury et al., 2016; Najman et al., 2017). Both sources of prenatal stress have also been associated with alterations to the hypothalamic-pituitary-adrenal (HPA) axis (Osborne et al., 2018; Van den Bergh et al., 2008), increased inflammation (Plant et al., 2016), and hyperresponsivity in the amygdala in the offspring (Knaap et al., 2018), offering support for a programming effect of prenatal stress on offspring depression through biologically mediated mechanisms (Hantsoo et al., 2019; Kim et al., 2015).

There is also evidence for the continuity of prenatal maternal depression into the postnatal period (Barker et al., 2011; Lereya and Wolke, 2013). Both pre- and postnatal maternal depression are also independently associated with emotional problems in childhood and psychopathology in adulthood, with postnatal depression proposed to act via environmental influences such as altered parenting behaviours or family environments (Munhoz et al., 2017; Pearson et al., 2013; Rees et al., 2019). Indicators of family adversity such as social and economic difficulties have also been shown to have a moderate persistence from the prenatal to postnatal period (Lereya and Wolke, 2013). Considering that both sources of stress continue into the postnatal period, it is important to account for these in order to examine the effect of prenatal stress independently from postnatal influences.

These early sources of stress may continue to exert influences throughout the developmental periods. However, periods beyond the early postnatal years are rarely examined when investigating the longitudinal association between prenatal stress and offspring depression. One of the most consistent childhood risk factors for depression is exposure to trauma (Copeland et al., 2018), which can lead to similar alterations to biological systems implicated in depression (Cattaneoet al., 2015). The evidence on childhood trauma is especially strong for caregiver-inflicted trauma, such as emotional, physical and sexual abuse, with lasting effect into adulthood (Lindert et al., 2014; Mandelli et al., 2015). Peer bullying has also been associated with increased risk of depression, even after controlling for childhood psychiatric disorders (Copeland et al., 2013) and genetic liability (Singham et al., 2017), consistent with a causal effect (Moore et al., 2017). As well as being one of the most consistent predictors of depression and other psychopathologies (Sahle et al., 2021; Moore et al., 2017), there is also evidence that childhood trauma may mediate the association between indicators of biological risk and emotional and psychiatric problems such as psychosis (Liu et al., 2020, 2019; Wolke et al., 2015), suggesting that childhood trauma may not just be a risk factor for psychopathologies, but can also be a consequence of earlier experiences.

Indeed, prenatal maternal depression has been associated with increased exposure to childhood trauma, possibly due to poorer attachment, maladaptive parenting and a programming effect of prenatal stress on offspring temperament (Azeredo et al., 2017; Lereya and Wolke, 2013; Pawlby et al., 2011). An indirect pathway has been found from maternal prenatal depression to offspring depression in adulthood via increased exposure to childhood trauma (Plant et al., 2015). However, sample size was small (*N* = 103) and specific trauma types were not examined. Furthermore, given the moderate heritability of depression (Flint and Kendler, 2014), part of the association between prenatal maternal depression and offspring depression may be explained by genetic liability, which should be controlled for. Lastly, family adversity was not examined independently from maternal depression when testing the mediating effect of childhood trauma, thus it would be important to investigate whether the same pathways are found from social/economic stress as well as psychological stress.

The aim of this prospective longitudinal study was to the examine the direct effects of pre- and postnatal stress, as indicated by maternal depression and family adversity, and their indirect effects via childhood trauma on offspring depression in adulthood. In a large population-based sample followed up from pregnancy to 24 years, we first investigated the individual effects of pre- and postnatal stress and childhood trauma on offspring depression at 24 years. Secondly, we examined childhood trauma as a mediator in the pathway from pre- and postnatal stress to offspring depression.

## 2 Methods

### 2.1 Sample

The sample was drawn from the ALSPAC cohort, a prospective population study of 14 541 pregnant women who resided in the region of Avon, Southwest of England, with expected delivery dates between April 1, 1991 to December 31, 1992, and has been described previously (Boyd et al., 2013; Fraser et al., 2013; Northstone et al., 2019). In total, 3506 participants who attended clinical assessment at 24 years were included. A fully searchable data dictionary and variable search tool can be found on the study website (http://www.bristol.ac.uk/alspac/researchers/our-data). Study data were collected and managed using REDCap (Research Electronic Data Capture) electronic data capture tools hosted at the University of Bristol, a secure, web-based software platform designed to support data capture for research studies (Harris et al., 2009). Ethical approval was obtained from the ALSPAC Ethics and Law Committee and the Local Research Ethics Committees. Informed consent for the use of data collected via questionnaires and clinics was obtained from participants and parents following the recommendations of the ALSPAC Ethics and Law Committee at the time.

### 2.2 Measures

#### 2.2.1 Depression at 24 years

Participants attended a study clinic at 24 years and the Computerized Interview Schedule-Revised (CIS-R) was used to derive diagnosis for depression based on the ICD-10 criteria (Bell et al., 2005; Patton et al., 1999). It is a self-administered computerized interview and is the standardized tool for assessing common mental health disorders (Bell et al., 2005; Patton et al., 1999). Severity of depression was categorized into mild, moderate and severe according to symptoms experienced in the past two weeks, using the ICD-10 criteria. The outcome of interest in the current study was a binary variable indicating no depression diagnosis or any depression diagnosis (mild, moderate or severe) (Dantchev et al., 2019).

#### 2.2.2 Maternal depression

Maternal depression was assessed using the Edinburgh Postnatal Depression Scale (EPDS), a 10-item self-reported depression questionnaire that is well validated for use during pregnancy and in the post-partum period (Cox et al., 1987). Each item was scored from 0 to 3 and referred to feelings over the past week. Traditionally a cut-off score of 13 or more has been used to indicate clinically significant symptoms (Hewitt et al., 2009; Matthey et al., 2006), however in the current study continuous scores are used to take into account subtle variations in symptoms. EPDS scores are averaged across two periods during pregnancy (at 18 and 32 weeks) and three periods postnatally (2, 8 and 21 months) to indicate prenatal and postnatal maternal depression.

#### 2.2.3 Family adversity

Family adversity was measured during pregnancy and in the postnatal period using the long version of the Family Adversity Index (FAI), a cumulative index developed from the ALSPAC data based on Rutter's indicators of adversity (Steer et al., 2004). The original long index is comprised of 18 items including age of mother, housing situation, educational qualifications, financial situation, relationship with partner, family characteristics, social network, substance abuse, criminal behaviours and maternal psychopathology (Bowen et al., 2005). As maternal depression was investigated as a separate source of stress in the current study, the scale of maternal psychopathology was removed from the FAI to prevent over-controlling for the effect of maternal mental health. Thus, the FAI index comprised of 17 items and ranged from 0 to 17. Prenatal FAI indicates adversities experienced between 8- and 32-weeks’ gestation, and postnatal FAI indicates adversities between 0 and 2 years of age. The distribution of the FAI within the ALSPAC cohort showed a non-normal, positively skewed distribution, and so was categorised into no adversity (score of 0), few adversities (score of 1,2), and many adversities (score of 3 or more).

#### 2.2.4 Trauma

Childhood trauma experienced up to 17 years was derived in a previous study from 121 questions completed by either parents or participants on the frequency and severity of caregiver-inflicted trauma (types: physical abuse, emotional abuse, sexual abuse, emotional neglect, domestic violence) and peer bullying (Croft et al., 2018). All trauma assessments up to 5 years were reported by parents, a mixture of parent and child reports were used between 5 and 11 years, and child report was predominantly used between 11 and 17 years. A detailed description of the trauma measure has been described previously (Croft et al., 2018). A composite measure of exposure to any trauma (caregiver or peer inflicted) was derived from these individual trauma exposures, and categorized into no exposure, exposure to one trauma, and exposure to two or more traumas. Specific trauma types were assessed in a sensitivity analysis.

#### 2.2.5 Other control variables

Sex of the participant was coded as male or female at birth. Polygenic risk scores indexing the participants’ cumulative genetic vulnerability for major depressive disorder (MDD) and neuroticism were derived from genome-wide association study (GWAS), using summary statistics from discovery studies (23andMe and UK Biobank), which has been reported previously (Jones et al., 2018). Scores were standardized using a list of single nucleotide polymorphisms (SNPs) associated with these outcomes in the discovery samples at a p-threshold of 0.05 (Jones et al., 2018).

### 2.3 Statistical analysis

All analyses were conducted in R version 3.6.3. Simple logistic regression models were first used to examine the individual effect of each risk factor (prenatal and postnatal maternal depression, prenatal and postnatal FAI, childhood trauma) on offspring depression. Ordinal logistic regression models also examined the association between each pre- and postnatal risk and childhood trauma. Childhood trauma was coded as an ordered variable with linear terms, and proportional odds assumption was not violated.

Path analysis was used to estimate direct and indirect effects (product of coefficients method) from pre- and postnatal stress via childhood trauma to depression at 24 years, using the “semTools” package. Simple path models were first examined from each of the four indicators of pre- and postnatal stress to offspring depression via childhood trauma. These four pathways were then modeled simultaneously in one path model, controlling for the effects of covariates. Missing data on predictor variables were handled using multivariate imputation by chained equations (“mice” package) with 40 imputed datasets. Data were imputed up to the total sample with complete data on depression at 24 years (*N* = 3506) using all predictors included in the analysis. Standardised path coefficients and 95% confidence intervals are reported.

### 2.4 Sensitivity analysis

We further examined the indirect effect of each specific trauma in separate path models. The same model was specified as above, with exposure to childhood trauma replaced by exposure to each specific trauma, and all paths from pre- and postnatal stress were modeled simultaneously.

## 3 Results

### 3.1 Sample characteristics

Characteristics of people lost to attrition have been reported previously in this cohort, with those dropping out more likely to be from households with financial difficulties, lower educational qualifications, poor housing and of mothers who were more likely to have experienced psychopathology during pregnancy (Wolke et al., 2009). The proportion of missing data in the current sample ranged from 0.2% to 25% (see supplementary materials, Table S1 for number of missing cases for each predictor).

The majority of participants were female (62.4%). More family adversities were reported during the postnatal period compared to prenatal period (19.5% vs 8.1% who experienced 3 or more adversities), and 29% reported multiple trauma exposure (exposed to two or more trauma) up to 17 years. The prevalence of depression was 10.8% at 24 years (Table 1), consistent with previous reports (Dantchev et al., 2019; Fernandes et al., 2020).

**Table 1:** Sample characteristics (*N* = 3506).

	N	%
**Sex (Female)**	2186	62.4
**Prenatal FAI** (*N* = 3390)		
1–2 adversities	1164	34.3
Three or more adversities	273	8.1
**Postnatal FAI** (*N* = 3428)		
1–2 adversities	1539	44.9
Three or more adversities	667	19.5
**Childhood trauma** (*N* = 3500)		
One trauma	1023	29.2
Two or more trauma	1016	29.0
**Depression at 24**	379	10.8
	**Mean**	**SD**
**Prenatal maternal depression** (*N* = 3404)	6.45	4.26
**Postnatal maternal depression** (*N* = 3427)	5.48	4.02
**Standardised genetic risk score for MDD** (*N* = 2625)	−0.01	1.01
**Standardised genetic risk score for Neuroticism** (*N* = 2625)	−0.04	1.00

### 3.2 Risk factors for offspring depression

Simple logistic regression models showed that all risk factors (prenatal and postnatal maternal depression, prenatal and postnatal FAI, childhood trauma) were associated with offspring depression at 24 years. Childhood trauma was associated with the highest odds of offspring depression (Table 2).

**Table 2:** Simple logistic regressions on the effect of pre- and postnatal maternal depression/FAI and childhood trauma on depression at 24 years (Multiple imputation, *N* = 3506).

	Depression		
	OR	95% CI	*p*-value
**Prenatal maternal depression**^a^	1.05	1.02–1.08	< 0.001
**Postnatal maternal depression**^a^	1.04	1.01–1.07	0.002
**Prenatal FAI**	1.53	1.19–1.97	0.001
**Postnatal FAI**	1.49	1.21–1.83	< 0.001
**Childhood trauma**	1.73	1.46–2.07	< 0.001

a Effect is associated with each increased score in the Edinburgh Postnatal Depression Scale.

### 3.3 Risk factors for childhood trauma

Both maternal depression and family adversity during the pre- and postnatal periods were associated with increased odds of childhood trauma (Table 3). Each increased point in mothers’ depression score during the pre- and postnatal period was associated with 6 and 7% odds of increased exposure to childhood trauma, respectively. Increased exposure to family adversity during the pre- and postnatal period was also associated with increased exposure to childhood trauma (Table 3).

**Table 3:** Simple ordinal logistic regressions on the effect of pre- and postnatal maternal depression/FAI on childhood trauma (Multiple imputation, *N* = 3506).

	Childhood trauma		
	OR	95% CI	*p*-value
**Prenatal maternal depression**^a^	1.06	1.05–1.07	< 0.001
**Postnatal maternal depression**^a^	1.07	1.05–1.08	< 0.001
**Prenatal FAI**	1.75	1.58–1.94	< 0.001
**Postnatal FAI**	1.78	1.65–1.92	< 0.001

a Effect is associated with each increased score in the Edinburgh Postnatal Depression Scale.

### 3.4 Path analysis

**Fig. 1:**
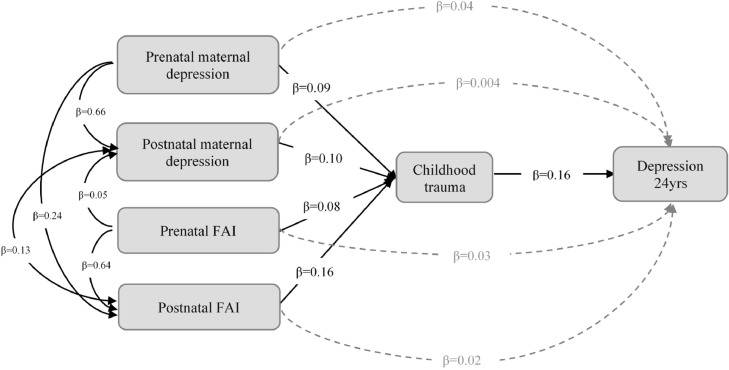
Indirect effects from pre- and postnatal maternal depression/FAI to depression at 24 years via childhood trauma. Standardised path estimates are shown, and all paths were modelled simultaneously, controlling for sex, genetic risk for MDD and neuroticism.

Simple path analyses found direct and indirect effects of prenatal maternal depression and prenatal FAI on offspring depression via childhood trauma (supplementary materials, Tables S2–S5). Indirect effects were also found from postnatal maternal depression and postnatal FAI via childhood trauma, but there was weaker evidence for their direct effects. When all pathways were modeled simultaneously and adjusted for control variables, no direct effects were found from pre- and postnatal maternal depression and FAI. However, indirect pathways were found from all four indicators of pre- and postnatal stress to offspring depression via childhood trauma (Table 4, Fig. 1). The strongest indirect pathway was from postnatal FAI via childhood trauma, which mediated 16% of the total effect of pre- and postnatal stress on offspring depression. This was calculated by dividing the indirect effect by the total effect. Other indirect pathways (from pre- and postnatal maternal depression, prenatal FAI) via childhood trauma mediated between 7 and 10% of the total effect on offspring depression at 24 years. Being female (SE = 0.18, 95%CI: 0.12–0.24) and polygenic risk score for MDD (SE = 0.10, 95%CI: 0.04–0.16) were also associated with increased risk of depression.

**Table 4:** Path analysis showing the direct and indirect pathways from pre- and postnatal maternal depression/FAI to depression at 24 years via childhood trauma (Multiple imputation, *N* = 3506).

	SE	95%CI	*p*-value
**Depression**^a^**∼**			
Prenatal maternal depression	0.04	−0.04–0.12	0.338
Postnatal maternal depression	0.004	−0.07–0.08	0.912
Prenatal FAI	0.03	−0.04–0.11	0.401
Postnatal FAI	0.02	−0.05–0.09	0.595
Childhood trauma	**0.16**	**0.10–0.23**	**< 0.001**
**Childhood trauma**^a^**∼**			
Prenatal maternal depression	**0.09**	**0.04–0.14**	**0.001**
Postnatal maternal depression	**0.10**	**0.05–0.16**	**< 0.001**
Prenatal FAI	**0.08**	**0.03–0.13**	**0.002**
Postnatal FAI	**0.16**	**0.11–0.20**	**< 0.001**
**Postnatal FAI∼**			
Prenatal FAI	**0.64**	**0.59–0.68**	**< 0.001**
Prenatal maternal depression	**0.24**	**0.20–0.29**	**< 0.001**
**Postnatal maternal depression∼**			
Prenatal FAI	**0.05**	**0.03–0.08**	**< 0.001**
Prenatal maternal depression	**0.66**	**0.64–0.69**	**< 0.001**
**Covariance**			
Postnatal FAI ∼∼ Postnatal maternal depression	**0.13**	**0.10–0.15**	**< 0.001**
**Indirect effect**^a^			
Prenatal maternal depression → Childhood trauma → Depression	**0.015**	**0.004–0.025**	**0.008**
Postnatal maternal depression → Childhood trauma → Depression	**0.017**	**0.006–0.027**	**0.002**
Prenatal FAI → Childhood trauma → Depression	**0.012**	**0.003–0.022**	**0.012**
Postnatal FAI → Childhood trauma → Depression	**0.026**	**0.013–0.038**	**< 0.001**
**Total effect**	**0.163**	**0.091–0.236**	**< 0.001**

a Pathways modeled simultaneously, and controlled for sex, genetic risk score for MDD and neuroticism. Significant confounders: sex (female) and genetic risk score for MDD on depression, and sex (male) and genetic risk for neuroticism on trauma.

### 3.5 Sensitivity analysis

Indirect pathways were found via physical abuse, emotional abuse and peer bullying when specific trauma types were examined (supplementary materials, Tables S6–S11). Physical abuse mediated the association between postnatal FAI and offspring depression and accounted for 14% of the total effect. Emotional abuse mediated the association between pre- and postnatal maternal depression as well as postnatal FAI on offspring depression, accounting for 9 to 17% of the total effect. Peer bullying further mediated the association between prenatal maternal depression and offspring depression and accounted for 6% of the total effect.

## 4 Discussion

The current study investigated the longitudinal association between pre- and postnatal stress (maternal depression and family adversity), childhood trauma and offspring depression in adulthood. Although both sources of pre- and postnatal stress were associated with offspring depression, when all pathways were simultaneously modeled, their direct effects were attenuated, and only indirect pathways were found from each of them to offspring depression via increased exposure to childhood trauma.

Maternal prenatal depression was initially associated with increased risk of depression in the offspring, consistent with previous research and the programming model (Kim et al., 2015; Plant et al., 2015; Rogers et al., 2020). Every increased score in mother's depressive symptoms was associated with a 5% increased odds of offspring depression at 24 years, and both direct and indirect effects were found from prenatal maternal depression to offspring depression via childhood trauma. However, this direct effect was reduced once other pathways from postnatal maternal depression and FAI were controlled for, and only childhood trauma was directly associated with offspring depression. This is consistent with previous findings (Plant et al., 2015), and suggest that part of the effect of prenatal maternal depression may be attributed to a continuity of depression in the postnatal period as well as its association with family adversity, which has been shown previously (Najman et al., 2017).

Childhood trauma has also been consistently associated with risk of depression in adulthood in previous research (Copeland et al., 2018; Sahle et al., 2021), with recent evidence suggesting a causal link which has also been extended to other mental health disorders including psychosis (Croft et al., 2018; Warrier et al., 2021). With each additional trauma experienced, there was a 73% increased odds of depression at 24 years, and suggest a dose-response effect which has been reported previously (Copeland et al., 2018; Croft et al., 2018). Indirect pathways were found from all indicators of pre- and postnatal stress to offspring depression via increased exposure to childhood trauma. This is consistent with previous research on prenatal maternal depression (Plant et al., 2015) and extends this indirect pathway to family adversity as well, which has been shown previously to be associated with increased risk of childhood trauma (Conrad-Hiebner and Byram, 2020; Lereya and Wolke, 2013). These suggest potentially separate pathways from psychological stress and social/economic stress, involving both biological and environmental mechanisms. Furthermore, although the proportion of mediated effect varied, these findings consistently support the role of childhood trauma as an important environmental factor that should be routinely examined in longitudinal studies on the effects of prenatal stress on offspring depression.

Analysis of specific trauma revealed stronger evidence for indirect effects from postnatal FAI via physical abuse, and from postnatal maternal depression and FAI via emotional abuse. This suggests that environmental factors, such as increased harsh parenting and reduced emotional availability in mothers with depression and families with increased social and economic stress contributes to childhood trauma and offspring depression (Conrad-Hiebner and Byram, 2020; Conron et al., 2009; Kluczniok et al., 2016). Emotional abuse and peer bullying further mediated the association between prenatal maternal depression and offspring depression, even after accounting for postnatal influences. Biological mechanisms have been previously proposed, such as a programming effect of prenatal maternal depression on infant temperament and emotional reactivity, which may lead to maladaptive parenting and increase vulnerability to peer victimisation (Lereya and Wolke, 2013; O'Connor et al., 2003). Further investigation is needed to better understand biological and environmental mechanisms associated with different types of trauma.

The long-term consequences of early risk and childhood trauma may also be considered from a life history perspective (LHP), where experiences of neglect, abuse and peer trauma may herald a world where others cannot be trusted or are disappointing (Brüne, 2016; Del Giudice, 2014; Otto et al., 2021). Thus, LHP predicts a faster pace of life with physical and psychological resources expanded to allow to escape a family or context at an earlier age than those in non-abusive contexts. For example, it has been shown that puberty timing is earlier in those who experienced sexual abuse with the possibility of subsequent psychosocial difficulties (Noll et al., 2017). Depression is also associated with an increased risk of cardiovascular disease (Joynt et al., 2003), i.e. a potential trade off for faster living. Thus, depression may be understood as an adaptive response, or the adoption of certain life history strategies to compensate for the accumulation of adversity across the early stages of life. However, LHP or faster pacing of life after trauma including altered health behavior (earlier drinking, smoking) or alternative explanations requires comparative testing in further longitudinal study.

### 4.1 Strengths and limitations

The longitudinal nature of the study and prospectively measured data allowed the testing of pathways from pre- and postnatal stress to offspring depression in adulthood. The large sample size allowed investigation on the specificity of trauma in a sensitivity analysis, which extends previous research (Plant et al., 2015). The finding of a mediated pathway from prenatal maternal depression to offspring depression via childhood trauma further suggest the involvement of biological mechanisms, such as a programming effect, as some of the indirect effect remained even after postnatal influences were modeled simultaneously in one model. Lastly, the inclusion of family adversity allowed examination of social/economic sources of stress separately to maternal depression and suggest that both may contribute independently to offspring depression via childhood trauma.

Some limitations include the high attrition rate over a period of 24 years which is unavoidable in longitudinal studies, with those dropping out more likely to be from households with lower SES and increased maternal psychopathology, which in turn may also underestimate the prevalence of depression at 24 years (Wolke et al., 2009). However, it has been previously shown in simulations that selective dropout does not affect the validity of predictive associations (Wolke et al., 2009), although statistical power may be affected due to an underestimation in the prevalence rates. Secondly, the measure of trauma used in the current study included abuse or neglect from both the mother and partner (Croft et al., 2018). The differential effect of mother vs partner-inflicted abuse was not examined; thus it is possible that the association between maternal and offspring depression, and apparent mediation via childhood trauma, may be confounded by partner-inflicted abuse leading to depression in both the mother and offspring. The derived trauma variable also included both parent- and child-reported measures, which may introduce measurement error and underestimate the effect of trauma, especially as the proportion of child-reported trauma was roughly twice as high as parent-reported trauma (Croft et al., 2018). Thirdly, polygenic risk scores for MDD and neuroticism were only included in the current study as control variables. Polygenic risk scores account for more genetic variance compared to individual SNPs, and can be used as a proxy measure for genetic vulnerability to identify individuals at increased risk of depression (Crouch and Bodmer, 2020; Schoeler et al., 2019). However, they only explain a proportion of total genetic heritability, and are also associated with other psychiatric phenotypes apart from depression (Jones et al., 2018; Mistry et al., 2018). Lastly, there may be other indirect pathways or confounding variables that are associated with increased exposure to childhood trauma, such as childhood psychiatric problems and genetic risks for other mental health vulnerabilities not considered in this study (Schoeler et al., 2019).

## 5 Conclusion

The current study investigated the direct and indirect effects of pre- and postnatal stress on offspring depression in adulthood via childhood trauma. When all pathways were modeled simultaneously, no direct paths were found from pre- and postnatal maternal depression or family adversity to offspring depression, but all four indirect pathways were found via childhood trauma. This suggests that the risk attributed to pre- and postnatal stress can be partly explained by increased exposure to childhood trauma, which is a potentially modifiable factor. The mediating role of childhood trauma is also consistent with other areas of psychopathology, such as psychosis, contributing further to the research field that investigates childhood trauma as both an antecedent of psychopathologies as well as a consequence of early experiences. These findings further highlight the importance of interventions to reduce childhood trauma at home and at school, including improved access to interventions for pregnant mothers with depression, as well as increased family, social and school support for those at risk of economic and social adversity.

## Funding

Data analysis and manuscript writing was funded by a PhD studentship to YL at University of Warwick. JH, MH & SZ are supported by the 10.13039/100006662NIHR Biomedical Research Centre at University Hospitals Bristol and Weston NHS Foundation Trust and the University of Bristol. DW is further supported by European Union Horizon 2020 research and innovation programme (RECAP-preterm) under grant agreement: 733280. Collection of depression data was supported by 10.13039/501100007155MRC (Medical Research Council) grant to MH, grant number: MR/L022206/1. MRC and Wellcome Trust (Grant ref: 217065/Z/19/Z) and the University of Bristol provide core support for ALSPAC. GWAS data was generated by Sample Logistics and Genotyping Facilities at Wellcome Sanger Institute and LabCorp using support from 23andMe. A comprehensive list of grants is available at http://www.bristol.ac.uk/alspac/external/documents/grant-acknowledgements.pdf. The views expressed are those of the author(s) and not necessarily those of the NIHR or the Department of Health and Social Care. YL had full access to all the data used in this study and serves as guarantor for the contents of this paper.

## CRediT authorship contribution statement

**Yiwen Liu:** Conceptualization, Visualization, Data curation, Formal analysis, Writing – original draft, Formal analysis, Writing – review & editing. **Jon Heron:** Funding acquisition, Formal analysis, Writing – review & editing. **Matthew Hickman:** Funding acquisition, Investigation, Formal analysis, Writing – review & editing. **Stanley Zammit:** Conceptualization, Visualization, Funding acquisition, Formal analysis, Writing – review & editing. **Dieter Wolke:** Conceptualization, Visualization, Funding acquisition, Formal analysis, Writing – original draft, Writing – review & editing.

## Declaration of Competing Interest

None.

## Appendix Supplementary materials

Image, application 1

## References

[bib0001] Azeredo C.M., Santos I.S., Barros A.J.D., Barros F.C., Matijasevich A. (2017). Maternal depression and bullying victimization among adolescents: results from the 2004 Pelotas cohort study. Depress. Anxiety.

[bib0002] Barker D.J. (2007). The origins of the developmental origins theory. J. Intern. Med..

[bib0003] Barker E.D., Jaffee S.R., Uher R., Maughan B. (2011). The contribution of prenatal and postnatal maternal anxiety and depression to child maladjustment. Depress. Anxiety.

[bib0004] Bell T., Watson M., Sharp D., Lyons I., Lewis G. (2005). Factors associated with being a false positive on the general health questionnaire. Soc. Psychiatry Psychiatr. Epidemiol..

[bib0005] Bowen E., Heron J., Waylen A., Wolke D., the ALSPAC study team (2005). Domestic violence risk during and after pregnancy: findings from a British longitudinal study. BJOG: Int. J. Obstet. Gynaecol..

[bib0006] Boyd A., Golding J., Macleod J., Lawlor D.A., Fraser A., Henderson J., Molloy L., Ness A., Ring S., Davey Smith G. (2013). Cohort profile: the ‘children of the 90s’—the index offspring of the avon longitudinal study of parents and children. Int. J. Epidemiol..

[bib0007] Brüne M. (2016). Borderline personality disorder: why ‘fast and furious’?. Evol. Med. Public Health.

[bib0008] Cattaneo A., Macchi F., Plazzotta G., Veronica B., Bocchio-Chiavetto L., Riva M.A., Pariante C.M. (2015). Inflammation and neuronal plasticity: a link between childhood trauma and depression pathogenesis. Front. Cell. Neurosci..

[bib0009] Conrad-Hiebner A., Byram E. (2020). The temporal impact of economic insecurity on child maltreatment: a systematic review. Trauma Violence Abuse.

[bib0010] Conron K.J., Beardslee W., Koenen K.C., Buka S.L., Gortmaker S.L. (2009). A longitudinal study of maternal depression and child maltreatment in a national sample of families investigated by child protective services. Arch. Pediatr. Adolesc. Med..

[bib0011] Copeland W.E., Shanahan L., Hinesley J., Chan R.F., Aberg K.A., Fairbank J.A., Oord E.J.C.G., van den, Costello E.J. (2018). Association of childhood trauma exposure with adult psychiatric disorders and functional outcomes. JAMA Netw. Open.

[bib0012] Copeland W.E., Wolke D., Angold A., Costello E.J. (2013). Adult psychiatric outcomes of bullying and being bullied by peers in childhood and adolescence. JAMA Psychiatry.

[bib0013] Cox J.L., Holden J.M., Sagovsky R. (1987). Detection of postnatal depression. development of the 10-item edinburgh postnatal depression scale. Br. J. Psychiatry.

[bib0014] Croft J., Heron J., Teufel C., Cannon M., Wolke D., Thompson A., Houtepen L., Zammit S. (2018). Association of trauma type, age of exposure, and frequency in childhood and adolescence with psychotic experiences in early adulthood. JAMA Psychiatry.

[bib0015] Crouch D.J.M., Bodmer W.F. (2020). Polygenic inheritance, GWAS, polygenic risk scores, and the search for functional variants. Proc. Natl. Acad. Sci. U. S. A..

[bib0016] Dantchev S., Hickman M., Heron J., Zammit S., Wolke D. (2019). The independent and cumulative effects of sibling and peer bullying in childhood on depression, anxiety, suicidal ideation, and self-harm in adulthood. Front. Psychiatry.

[bib0017] Del Giudice M. (2014). An evolutionary life history framework for psychopathology. Psychol. Inq..

[bib0018] Fernandes G.S., Lewis G., Hammerton G., Abeysekera K., Mahedy L., Edwards A., Lewis G., Hickman M., Heron J. (2020). Alcohol consumption and internalising disorders in young adults of ALSPAC: a population-based study. J. Epidemiol. Commun. Health.

[bib0019] Flint J., Kendler K.S. (2014). The genetics of major depression. Neuron.

[bib0020] Fraser A., Macdonald-Wallis C., Tilling K., Boyd A., Golding J., Davey Smith G., Henderson J., Macleod J., Molloy L., Ness A., Ring S., Nelson S.M., Lawlor D.A. (2013). Cohort profile: the avon longitudinal study of parents and children: ALSPAC mothers cohort. Int. J. Epidemiol..

[bib0021] Hantsoo L., Kornfield S., Anguera M.C., Epperson C.N. (2019). Inflammation: a proposed intermediary between maternal stress and offspring neuropsychiatric risk. Biol. Psychiatry.

[bib0022] Harris P.A., Taylor R., Thielke R., Payne J., Gonzalez N., Conde J.G. (2009). Research electronic data capture (REDCap)–a metadata-driven methodology and workflow process for providing translational research informatics support. J. Biomed. Inform..

[bib0023] Hewitt C., Gilbody S., Brealey S., Paulden M., Palmer S., Mann R., Green J., Morrell J., Barkham M., Light K., Richards D. (2009). Methods to identify postnatal depression in primary care: an integrated evidence synthesis and value of information analysis. Health Technol. Assess..

[bib0024] Jones H.J., Heron J., Hammerton G., Stochl J., Jones P.B., Cannon M., Smith G.D., Holmans P., Lewis G., Linden D.E.J., O'Donovan M.C., Owen M.J., Walters J., Zammit S. (2018). Investigating the genetic architecture of general and specific psychopathology in adolescence. Transl. Psychiatry.

[bib0025] Joynt K.E., Whellan D.J., O'Connor C.M. (2003). Depression and cardiovascular disease: mechanisms of interaction. Biol. Psychiatry.

[bib0026] Kim D.R., Bale T.L., Epperson C.N. (2015). Prenatal programming of mental illness: current understanding of relationship and mechanisms. Curr. Psychiatry Rep..

[bib0027] Kingsbury M., Weeks M., MacKinnon N., Evans J., Mahedy L., Dykxhoorn J., Colman I. (2016). Stressful life events during pregnancy and offspring depression: evidence from a prospective cohort study. J. Am. Acad. Child Adolesc. Psychiatry.

[bib0028] Kluczniok D., Boedeker K., Fuchs A., Attar C.H., Fydrich T., Fuehrer D., Dittrich K., Reck C., Winter S., Heinz A., Herpertz S.C., Brunner R., Bermpohl F. (2016). Emotional availability in mother–child interaction: the effects of maternal depression in remission and additional history of childhood abuse. Depress. Anxiety.

[bib0029] Knaap N.J.F., van der Klumpers F., Marroun H.E., Mous S., Schubert D., Jaddoe V., Hofman A., Homberg J.R., Tiemeier H., White T., Fernández G. (2018). Maternal depressive symptoms during pregnancy are associated with amygdala hyperresponsivity in children. Eur. Child Adolesc. Psychiatry.

[bib0030] Lereya S.T., Wolke D. (2013). Prenatal family adversity and maternal mental health and vulnerability to peer victimisation at school: prenatal family stressors and vulnerability to peer victimisation. J. Child Psychol. Psychiatry.

[bib0031] Lewis A., Austin E., Knapp R., Vaiano T., Galbally M. (2015). Perinatal maternal mental health, fetal programming and child development. Healthcare.

[bib0032] Lindert J., von Ehrenstein O.S., Grashow R., Gal G., Braehler E., Weisskopf M.G. (2014). Sexual and physical abuse in childhood is associated with depression and anxiety over the life course: systematic review and meta-analysis. Int. J. Public Health.

[bib0033] Liu Y., Mendonça M., Cannon M., Jones P.B., Lewis G., Thompson A., Zammit S., Wolke D. (2020). Testing the independent and joint contribution of exposure to neurodevelopmental adversity and childhood trauma to risk of psychotic experiences in adulthood. Schizophr Bull.

[bib0034] Liu Y., Mendonça M., Johnson S., O'Reilly H., Bartmann P., Marlow N., Wolke D. (2019). Testing the neurodevelopmental, trauma and developmental risk factor models of psychosis using a naturalistic experiment. Psychol. Med..

[bib0035] Mandelli L., Petrelli C., Serretti A. (2015). The role of specific early trauma in adult depression: a meta-analysis of published literature. Childhood trauma and adult depression. Eur. Psychiatry.

[bib0036] Matthey S., Henshaw C., Elliott S., Barnett B. (2006). Variability in use of cut-off scores and formats on the edinburgh postnatal depression scale: implications for clinical and research practice. Arch. Womens Ment. Health.

[bib0037] Mistry S., Harrison J.R., Smith D.J., Escott-Price V., Zammit S. (2018). The use of polygenic risk scores to identify phenotypes associated with genetic risk of bipolar disorder and depression: a systematic review. J. Affect Disord..

[bib0038] Moore S.E., Norman R.E., Suetani S., Thomas H.J., Sly P.D., Scott J.G. (2017). Consequences of bullying victimization in childhood and adolescence: a systematic review and meta-analysis. World J. Psychiatry.

[bib0039] Munhoz T.N., Santos I.S., Barros A.J.D., Anselmi L., Barros F.C., Matijasevich A. (2017). Perinatal and postnatal risk factors for disruptive mood dysregulation disorder at age 11: 2004 pelotas birth cohort study. J. Affect Disord..

[bib0040] Najman J.M., Plotnikova M., Williams G.M., Alati R., Mamun A.A., Scott J., Clavarino A.M., Wray N. (2017). Maternal depression and family adversity: linked pathways to offspring depression?. J. Psychiatr Res..

[bib0041] Noll J.G., Trickett P.K., Long J.D., Negriff S., Susman E.J., Shalev I., Li J.C., Putnam F.W. (2017). Childhood sexual abuse and early timing of puberty. J. Adolesc. Health.

[bib0042] Northstone K., Lewcock M., Groom A., Boyd A., Macleod J., Timpson N., Wells N. (2019). The avon longitudinal study of parents and children (ALSPAC): an update on the enrolled sample of index children in 2019. Wellcome Open Res..

[bib0043] O'Connor T.G., Heron J., Golding J., Glover V., ALSPAC Study Team (2003). Maternal antenatal anxiety and behavioural/emotional problems in children: a test of a programming hypothesis. J. Child Psychol. Psychiatry.

[bib0044] O'Donnell K.J., Meaney M.J. (2016). Fetal origins of mental health: the developmental origins of health and disease hypothesis. AJP.

[bib0045] Osborne S., Biaggi A., Chua T.E., Du Preez A., Hazelgrove K., Nikkheslat N., Previti G., Zunszain P.A., Conroy S., Pariante C.M. (2018). Antenatal depression programs cortisol stress reactivity in offspring through increased maternal inflammation and cortisol in pregnancy: the psychiatry research and motherhood – depression (PRAM-D) Study. Psychoneuroendocrinology.

[bib0046] Otto B., Kokkelink L., Brüne M. (2021). Borderline personality disorder in a “life history theory” perspective: evidence for a fast “pace-of-life-syndrome. Front. Psychol..

[bib0047] Patton G.C., Coffey C., Posterino M., Carlin J.B., Wolfe R., Bowes G. (1999). A computerised screening instrument for adolescent depression: population-based validation and application to a two-phase case-control study. Soc. Psychiatry Psychiatr Epidemiol..

[bib0048] Pawlby S., Hay D., Sharp D., Waters C.S., Pariante C.M. (2011). Antenatal depression and offspring psychopathology: the influence of childhood maltreatment. Br. J. Psychiatry.

[bib0049] Pearson R.M., Evans J., Kounali D., Lewis G., Heron J., Ramchandani P.G., O'Connor T.G., Stein A. (2013). Maternal depression during pregnancy and the postnatal period: risks and possible mechanisms for offspring depression at age 18 years. JAMA Psychiatry.

[bib0050] Plant D.T., Pariante C.M., Sharp D., Pawlby S. (2015). Maternal depression during pregnancy and offspring depression in adulthood: role of child maltreatment. Br. J. Psychiatry.

[bib0051] Plant D.T., Pawlby S., Sharp D., Zunszain P.A., Pariante C.M. (2016). Prenatal maternal depression is associated with offspring inflammation at 25 years: a prospective longitudinal cohort study. Transl. Psychiatry.

[bib0052] Räikkönen K., Pesonen A.K. (2009). Early life origins of psychological development and mental health. Scand. J. Psychol..

[bib0053] Rees S., Channon S., Waters C.S. (2019). The impact of maternal prenatal and postnatal anxiety on children's emotional problems: a systematic review. Eur. Child Adolesc. Psychiatry.

[bib0054] Rogers A., Obst S., Teague S.J., Rossen L., Spry E.A., Macdonald J.A., Sunderland M., Olsson C.A., Youssef G., Hutchinson D. (2020). Association between maternal perinatal depression and anxiety and child and adolescent development: a meta-analysis. JAMA Pediatr..

[bib0055] Sahle B.W., Reavley N.J., Li W., Morgan A.J., Yap M.B.H., Reupert A., Jorm A.F. (2021). The association between adverse childhood experiences and common mental disorders and suicidality: an umbrella review of systematic reviews and meta-analyses. Eur. Child Adolesc. Psychiatry.

[bib0056] Schoeler T., Choi S.W., Dudbridge F., Baldwin J., Duncan L., Cecil C.M., Walton E., Viding E., McCrory E., Pingault J.B. (2019). Multi–polygenic score approach to identifying individual vulnerabilities associated with the risk of exposure to bullying. JAMA Psychiatry.

[bib0057] Singham T., Viding E., Schoeler T., Arseneault L., Ronald A., Cecil C.M., McCrory E., Rijsdijk F., Pingault J.B. (2017). Concurrent and longitudinal contribution of exposure to bullying in childhood to mental health. JAMA Psychiatry.

[bib0058] Steer C., Wolke D., the ALSPAC study team (2004). Proceedings of the Presented at the Poster presentation. 3rd Conference of Epidemiological Longitudinal Studies in Europe.

[bib0059] Tirumalaraju V., Suchting R., Evans J., Goetzl L., Refuerzo J., Neumann A., Anand D., Ravikumar R., Green C.E., Cowen P.J., Selvaraj S. (2020). Risk of depression in the adolescent and adult offspring of mothers with perinatal depression. JAMA Netw. Open.

[bib0060] Van den Bergh B.R.H., Calster B.V., Smits T., Huffel S.V., Lagae L. (2008). Antenatal maternal anxiety is related to HPA-axis dysregulation and self-reported depressive symptoms in adolescence: a prospective study on the fetal origins of depressed mood. Neuropsychopharmacol.

[bib0061] Van den Bergh B.R.H., van den Heuvel M.I., Lahti M., Braeken M., de Rooij S.R., Entringer S., Hoyer D., Roseboom T., Räikkönen K., King S., Schwab M. (2017). Prenatal developmental origins of behavior and mental health: the influence of maternal stress in pregnancy. Neurosci. Biobehav. Rev..

[bib0062] Warrier V., Kwong A.S.F., Luo M., Dalvie S., Croft J., Sallis H.M., Baldwin J., Munafò M.R., Nievergelt C.M., Grant A.J., Burgess S., Moore T.M., Barzilay R., McIntosh A., van IJzendoorn M.H., Cecil C.A.M. (2021). Gene–environment correlations and causal effects of childhood maltreatment on physical and mental health: a genetically informed approach. Lancet Psychiatry.

[bib0063] Wolke D., Baumann N., Strauss V., Johnson S., Marlow N. (2015). Bullying of preterm children and emotional problems at school age: cross-culturally invariant effects. J. Pediatr..

[bib0064] Wolke D., Waylen A., Samara M., Steer C., Goodman R., Ford T., Lamberts K. (2009). Selective drop-out in longitudinal studies and non-biased prediction of behaviour disorders. Br. J. Psychiatry.

