# Supplementary material for: Prenatal stress and offspring depression in adulthood: The mediating role of childhood trauma
Source: J Affect Disord. 2022 Jan 15;297:45–52. doi: 10.1016/j.jad.2021.10.019 (PMC8641663; doi:10.1016/j.jad.2021.10.019)
Supplement: Supplementary file 1 [file mmc1.docx]

**Supplementary Materials**

**Table S1.** Number of missing cases

| **Variable** | ***N* (%)** |
| --- | --- |
| Prenatal maternal depression | 102 (2.9%) |
| Postnatal maternal depression | 79 (2.3%) |
| Prenatal FAI | 116 (3.3%) |
| Postnatal FAI | 78 (2.2%) |
| Childhood trauma | 6 (0.2%) |
| Polygenic risk score (MDD) | 881 (25.1%) |
| Polygenic risk score (Neuroticism) | 881 (25.1%) |

**Table S2.** Unadjusted pathway from prenatal maternal depression to depression at 24 years via trauma

|  | **SE (95%CI)** | **p-value** |
| --- | --- | --- |
| **Depression ~** |  |  |
| Prenatal maternal depression | **0.06 (0.01 – 0.12)** | **0.023** |
| Trauma | **0.17 (0.11 – 0.23)** | **<0.001** |
| **Trauma ~** |  |  |
| Prenatal maternal depression | **0.25 (0.21 – 0.29)** | **<0.001** |
| **Indirect effect** |  |  |
| Prenatal maternal depression 🡪 Trauma 🡪 Depression | **0.04 (0.03 – 0.06)** | **<0.001** |
| **Total effect** | **0.11 (0.05 – 0.16)** | **<0.001** |

**Table S3**. Unadjusted pathway from postnatal maternal depression to depression at 24 years via trauma

|  | **SE (95%CI)** | **p-value** |
| --- | --- | --- |
| **Depression ~** |  |  |
| Postnatal maternal depression | 0.04 (-0.02 – 0.09) | 0.162 |
| Trauma | **0.18 (0.12 – 0.24)** | **<0.001** |
| **Trauma ~** |  |  |
| Postnatal maternal depression | **0.25 (0.21 – 0.29)** | **<0.001** |
| **Indirect effect** |  |  |
| Postnatal maternal depression 🡪 Trauma 🡪 Depression | **0.05 (0.03 – 0.06)** | **<0.001** |
| **Total effect** | **0.08 (0.03 – 0.14)** | **0.002** |

**Table S4**. Unadjusted pathway from prenatal FAI to depression at 24 years via trauma

|  | **SE (95%CI)** | **p-value** |
| --- | --- | --- |
| **Depression ~** |  |  |
| Prenatal FAI | **0.06 (0.003 – 0.11)** | **0.039** |
| Trauma | **0.18 (0.12 – 0.23)** | **<0.001** |
| **Trauma ~** |  |  |
| Prenatal FAI | **0.24 (0.20 – 0.28)** | **<0.001** |
| **Indirect effect** |  |  |
| Prenatal FAI 🡪 Trauma 🡪 Depression | **0.04 (0.03 – 0.06)** | **<0.001** |
| **Total effect** | **0.10 (0.05 – 0.15)** | **<0.001** |

**Table S5**. Unadjusted pathway from postnatal FAI to depression at 24 years via trauma

|  | **SE (95%CI)** | **p-value** |
| --- | --- | --- |
| **Depression ~** |  |  |
| Postnatal FAI | **0.06 (0.002 – 0.12)** | **0.043** |
| Trauma | **0.17 (0.11 – 0.23)** | **<0.001** |
| **Trauma ~** |  |  |
| Postnatal FAI | **0.28 (0.24 – 0.32)** | **<0.001** |
| **Indirect effect** |  |  |
| Postnatal FAI 🡪 Trauma 🡪 Depression | **0.05 (0.03 – 0.07)** | **<0.001** |
| **Total effect** | **0.11 (0.05 – 0.16)** | **<0.001** |

**Table S6**. Path analysis showing direct and indirect pathways from pre- and postnatal maternal depression/FAI to depression at 24 years via physical abuse.

|  | **SE (95%CI)** | **p-value** |
| --- | --- | --- |
| **Depression**^a^ **~** |  |  |
| Maternal depression (prenatal) | 0.04 (-0.04 – 0.12) | 0.291 |
| Maternal depression (postnatal) | 0.01 (-0.06 – 0.09) | 0.757 |
| FAI (prenatal) | 0.04 (-0.04 – 0.11) | 0.317 |
| FAI (postnatal) | 0.02 (-0.05 – 0.09) | 0.572 |
| Physical abuse | **0.21 (0.13 – 0.29)** | **<0.001** |
| **Physical abuse**^a^ **~** |  |  |
| Maternal depression (prenatal) | 0.05 (-0.01 – 0.12) | 0.118 |
| Maternal depression (postnatal) | 0.05 (-0.02 – 0.12) | 0.135 |
| FAI (prenatal) | 0.03 (-0.03 – 0.09) | 0.306 |
| FAI (postnatal) | **0.11 (0.05 – 0.17)** | **<0.001** |
| **Indirect effect**^a^ |  |  |
| Prenatal maternal depression 🡪 Physical abuse 🡪 Depression | 0.011 (-0.003 – 0.025) | 0.135 |
| Postnatal maternal depression 🡪 Physical abuse 🡪 Depression | 0.010 (-0.004 – 0.025) | 0.145 |
| Prenatal FAI 🡪 Physical abuse 🡪 Depression | 0.007 (-0.006 – 0.019) | 0.307 |
| Postnatal FAI 🡪 Physical abuse 🡪 Depression | **0.023 (0.008 – 0.037)** | **0.002** |
| **Total effect** | **0.164 (0.091 – 0.237)** | **<0.001** |

^a^All paths adjusted for each other, as well as for sex and genetic risk for MDD and neuroticism. Significant confounders: sex (female) and genetic risk score for MDD on depression. Additional pathways not shown here: from postnatal influences to prenatal influences, and covariance between postnatal maternal depression and postnatal FAI.

**Table S7**. Path analysis showing direct and indirect pathways from pre- and postnatal maternal depression/FAI to depression at 24 years via emotional abuse.

|  | **SE (95%CI)** | **p-value** |
| --- | --- | --- |
| **Depression**^a^ **~** |  |  |
| Maternal depression (prenatal) | 0.04 (-0.04 – 0.12) | 0.334 |
| Maternal depression (postnatal) | 0.00 (-0.08 – 0.08) | 0.998 |
| FAI (prenatal) | 0.04 (-0.04 – 0.11) | 0.307 |
| FAI (postnatal) | 0.02 (-0.06 – 0.09) | 0.694 |
| Emotional abuse | **0.16 (0.08 – 0.24)** | **<0.001** |
| **Emotional abuse**^a^ **~** |  |  |
| Maternal depression (prenatal) | **0.09 (0.03 – 0.16)** | **0.005** |
| Maternal depression (postnatal) | **0.15(0.08 – 0.21)** | **<0.001** |
| FAI (prenatal) | 0.04 (-0.02 – 0.10) | 0.203 |
| FAI (postnatal) | **0.18 (0.13 – 0.24)** | **<0.001** |
| **Indirect effect**^a^ |  |  |
| Prenatal maternal depression 🡪 Emotional abuse 🡪 Depression | **0.015 (0.002 – 0.027)** | **0.018** |
| Postnatal maternal depression 🡪 Emotional abuse 🡪 Depression | **0.023 (0.007 – 0.038)** | **0.004** |
| Prenatal FAI 🡪 Emotional abuse 🡪 Depression | 0.006 (-0.003 – 0.016) | 0.207 |
| Postnatal FAI 🡪 Emotional abuse 🡪 Depression | **0.028 (0.011 – 0.045)** | **0.001** |
| **Total effect** | **0.164 (0.091 – 0.237)** | **<0.001** |

^a^All paths adjusted for each other, as well as for sex and genetic risk for MDD and neuroticism. Significant confounders: sex (female) and genetic risk score for MDD on depression. Additional pathways not shown here: from postnatal influences to prenatal influences, and covariance between postnatal maternal depression and postnatal FAI.

**Table S8**. Path analysis showing direct and indirect pathways from pre- and postnatal maternal depression/FAI to depression at 24 years via sexual abuse.

|  | **SE (95%CI)** | **p-value** |
| --- | --- | --- |
| **Depression**^a^ **~** |  |  |
| Maternal depression (prenatal) | 0.04 (-0.04 – 0.12) | 0.291 |
| Maternal depression (postnatal) | 0.02 (-0.06 – 0.10) | 0.622 |
| FAI (prenatal) | 0.03 (-0.05 – 0.11) | 0.423 |
| FAI (postnatal) | 0.03 (-0.04 – 0.11) | 0.343 |
| Sexual abuse | **0.24 (0.15 – 0.33)** | **<0.001** |
| **Sexual abuse**^a^ **~** |  |  |
| Maternal depression (prenatal) | 0.04 (-0.04 – 0.13) | 0.280 |
| Maternal depression (postnatal) | 0.01 (-0.07 – 0.09) | 0.726 |
| FAI (prenatal) | 0.06 (-0.02 – 0.14) | 0.138 |
| FAI (postnatal) | 0.04 (-0.03 – 0.11) | 0.303 |
| **Indirect effect**^a^ |  |  |
| Prenatal maternal depression 🡪 Sexual abuse 🡪 Depression | 0.011 (-0.009 – 0.030) | 0.287 |
| Postnatal maternal depression 🡪 Sexual abuse 🡪 Depression | 0.003 (-0.015 – 0.022) | 0.721 |
| Prenatal FAI 🡪 Sexual abuse 🡪 Depression | 0.014 (-0.005 – 0.033) | 0.142 |
| Postnatal FAI 🡪 Sexual abuse 🡪 Depression | 0.009 (-0.008 – 0.026) | 0.309 |
| **Total effect** | **0.164 (0.091 – 0.236)** | **<0.001** |

^a^All paths adjusted for each other, as well as for sex and genetic risk for MDD and neuroticism. Significant confounders: sex (female) and genetic risk score for MDD on depression, and sex (female) on childhood sexual abuse. Additional pathways not shown here: from postnatal influences to prenatal influences, and covariance between postnatal maternal depression and postnatal FAI.

**Table S9**. Path analysis showing direct and indirect pathways from pre- and postnatal maternal depression/FAI to depression at 24 years via emotional neglect.

|  | **SE (95%CI)** | **p-value** |
| --- | --- | --- |
| **Depression**^a^ **~** |  |  |
| Maternal depression (prenatal) | 0.05 (-0.03 – 0.13) | 0.187 |
| Maternal depression (postnatal) | 0.03 (-0.05 – 0.10) | 0.515 |
| FAI (prenatal) | 0.03 (-0.04 – 0.11) | 0.387 |
| FAI (postnatal) | 0.04 (-0.04 – 0.11) | 0.316 |
| Emotional neglect | **0.11 (0.01 – 0.22)** | **0.040** |
| **Emotional neglect**^a^ **~** |  |  |
| Maternal depression (prenatal) | 0.003 (-0.08 – 0.09) | 0.946 |
| Maternal depression (postnatal) | -0.03 (-0.11 – 0.06) | 0.580 |
| FAI (prenatal) | **0.11 (0.02 – 0.19)** | **0.012** |
| FAI (postnatal) | 0.06 (-0.02 – 0.14) | 0.154 |
| **Indirect effect**^a^ |  |  |
| Prenatal maternal depression 🡪 Emotional neglect 🡪 Depression | 0.00 (-0.009 – 0.010) | 0.941 |
| Postnatal maternal depression 🡪 Emotional neglect 🡪 Depression | -0.003 (-0.012 – 0.007) | 0.562 |
| Prenatal FAI 🡪 Emotional neglect 🡪 Depression | 0.012 (-0.004 – 0.027) | 0.145 |
| Postnatal FAI 🡪 Emotional neglect 🡪 Depression | 0.007 (-0.003 – 0.016) | 0.158 |
| **Total effect** | **0.164 (0.091 – 0.237)** | **<0.001** |

^a^All paths adjusted for each other, as well as for sex and genetic risk for MDD and neuroticism. Significant confounders: sex (female) and genetic risk score for MDD on depression, and sex (male) on emotional neglect. Additional pathways not shown here: from postnatal influences to prenatal influences, and covariance between postnatal maternal depression and postnatal FAI.

**Table S10**. Path analysis showing direct and indirect pathways from pre- and postnatal maternal depression/FAI to depression at 24 years via domestic violence.

|  | **SE (95%CI)** | **p-value** |
| --- | --- | --- |
| **Depression**^a^ **~** |  |  |
| Maternal depression (prenatal) | 0.05 (-0.03 – 0.13) | 0.185 |
| Maternal depression (postnatal) | 0.02 (-0.05 – 0.10) | 0.562 |
| FAI (prenatal) | 0.05 (-0.03 – 0.12) | 0.236 |
| FAI (postnatal) | 0.04 (-0.03 – 0.12) | 0.268 |
| Domestic violence | -0.001 (-0.09 – 0.09) | 0.988 |
| **Domestic violence**^a^ **~** |  |  |
| Maternal depression (prenatal) | **0.12 (0.05 – 0.18)** | **0.001** |
| Maternal depression (postnatal) | **0.07 (0.01 – 0.14)** | **0.026** |
| FAI (prenatal) | **0.10 (0.04 – 0.17)** | **0.001** |
| FAI (postnatal) | **0.26 (0.21 – 0.32)** | **<0.001** |
| **Indirect effect**^a^ |  |  |
| Prenatal maternal depression 🡪 Domestic violence 🡪 Depression | 0.000(-0.010 – 0.010) | 0.988 |
| Postnatal maternal depression 🡪 Domestic violence 🡪 Depression | 0.00 (-0.007 - 0.007) | 0.989 |
| Prenatal FAI 🡪 Domestic violence 🡪 Depression | 0.00 (-0.009 – 0.009) | 0.986 |
| Postnatal FAI 🡪 Domestic violence 🡪 Depression | 0.00 (-0.023 – 0.023) | 0.988 |
| **Total effect** | **0.164 (0.091 – 0.237)** | **<0.001** |

^a^All paths adjusted for each other, as well as for sex and genetic risk for MDD and neuroticism. Significant confounder: sex (female) and genetic risk score for MDD on depression. Additional pathways not shown here: from postnatal influences to prenatal influences, and covariance between postnatal maternal depression and postnatal FAI.

**Table S11**. Path analysis showing direct and indirect pathways from pre- and postnatal maternal depression/FAI to depression at 24 years via peer bullying.

|  | **SE (95%CI)** | **p-value** |
| --- | --- | --- |
| **Depression**^a^ **~** |  |  |
| Maternal depression (prenatal) | 0.04 (-0.04 – 0.12) | 0.276 |
| Maternal depression (postnatal) | 0.02 (-0.06 – 0.10) | 0.611 |
| FAI (prenatal) | 0.04 (-0.03 – 0.12) | 0.247 |
| FAI (postnatal) | 0.04 (-0.03 – 0.11) | 0.275 |
| Peer bullying | **0.11 (0.03 – 0.18)** | **0.007** |
| **Peer bullying**^a^ **~** |  |  |
| Maternal depression (prenatal) | **0.09 (0.03 – 0.15)** | **0.005** |
| Maternal depression (postnatal) | 0.03 (-0.04 – 0.09) | 0.430 |
| FAI (prenatal) | 0.01 (-0.05 – 0.07) | 0.816 |
| FAI (postnatal) | 0.03 (-0.02 – 0.09) | 0.232 |
| **Indirect effect**^a^ |  |  |
| Prenatal maternal depression 🡪 Peer bullying 🡪 Depression | **0.010 (0.000 – 0.019)** | **0.040** |
| Postnatal maternal depression 🡪 Peer bullying 🡪 Depression | 0.003 (-0.004 – 0.009) | 0.432 |
| Prenatal FAI 🡪 Peer bullying 🡪 Depression | 0.001 (-0.005 – 0.007) | 0.815 |
| Postnatal FAI 🡪 Peer bullying 🡪 Depression | 0.004 (-0.003 – 0.010) | 0.260 |
| **Total effect** | **0.164 (0.090 – 0.236)** | **<0.001** |

^a^All paths adjusted for each other, as well as for sex and genetic risk for MDD and neuroticism. Significant confounders: sex (female) and genetic risk score for MDD on depression, and sex (male) on peer bullying. Additional pathways not shown here: from postnatal influences to prenatal influences, and covariance between postnatal maternal depression and postnatal FAI.
